# Supplementary material for: Altered neuronal start codon stringency favors cap-independent repeat-associated non-AUG translation
Source: Nucleic Acids Res. 2026 Sep 11;54(17):gkag880. doi: 10.1093/nar/gkag880 (PMC13563098; doi:10.1093/nar/gkag880)
Supplement: Supplementary file 1 [file gkag880_supplemental_file.pdf]

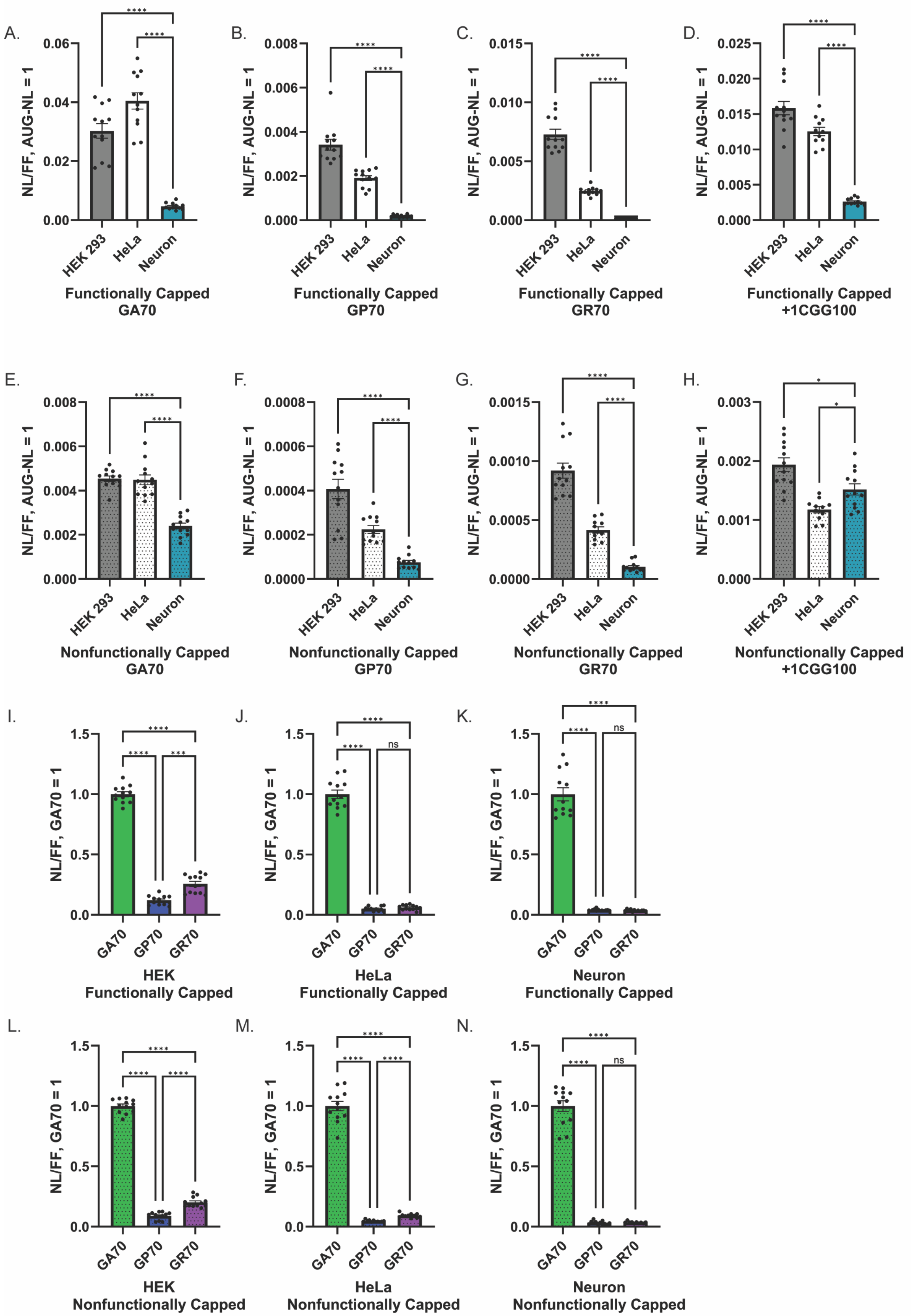

A.
B.
C.
D.
NL/FF, AUG-NL = 1
HEK 293
HeLa
Neuron
Functionally Capped GA70
Functionally Capped GP70
Functionally Capped GR70
Functionally Capped +1CGG100
E.
F.
G.
H.
Nonfunctionally Capped GA70
Nonfunctionally Capped GP70
Nonfunctionally Capped GR70
Nonfunctionally Capped +1CGG100
I.
J.
K.
NL/FF, GA70 = 1
GA70
GP70
GR70
ns
HEK Functionally Capped
HeLa Functionally Capped
Neuron Functionally Capped
L.
M.
N.
HEK Nonfunctionally Capped
HeLa Nonfunctionally Capped
Neuron Nonfunctionally Capped

Figure S1: Expression of RAN reporters differs by cell type. (A–H) Relative expression of RAN reporters in HEK293 cells, HeLa cells, and rat primary hippocampal neurons after transfection with functionally capped (A–D) or nonfunctionally capped (E–H) mRNA for 24 hours. All data is normalized to FF and graphed relative to functionally capped AUG-NL reporter expression, n = 12. (I–N) Relative expression of C9 RAN reporters in HEK293 cells, HeLa cells, and rat primary hippocampal neurons after transfection with functionally capped (I–K) or nonfunctionally capped (L–N) mRNA for 24 hours. Data is expressed as NL signal normalized to FF and relative to GA70 reporter expression, n = 12. Graphs represent mean ± SEM; Brown-Forsythe one-way ANOVA with Dunnet's multiple comparison correction. *P < 0.05, **P<0.01, ***P < 0.001, ****P < 0.0001. GA = poly-glycine-alanine, GP = poly-glycine-proline, GR = poly-glycine-arginine, +1CGG = poly-glycine, FF = firefly.

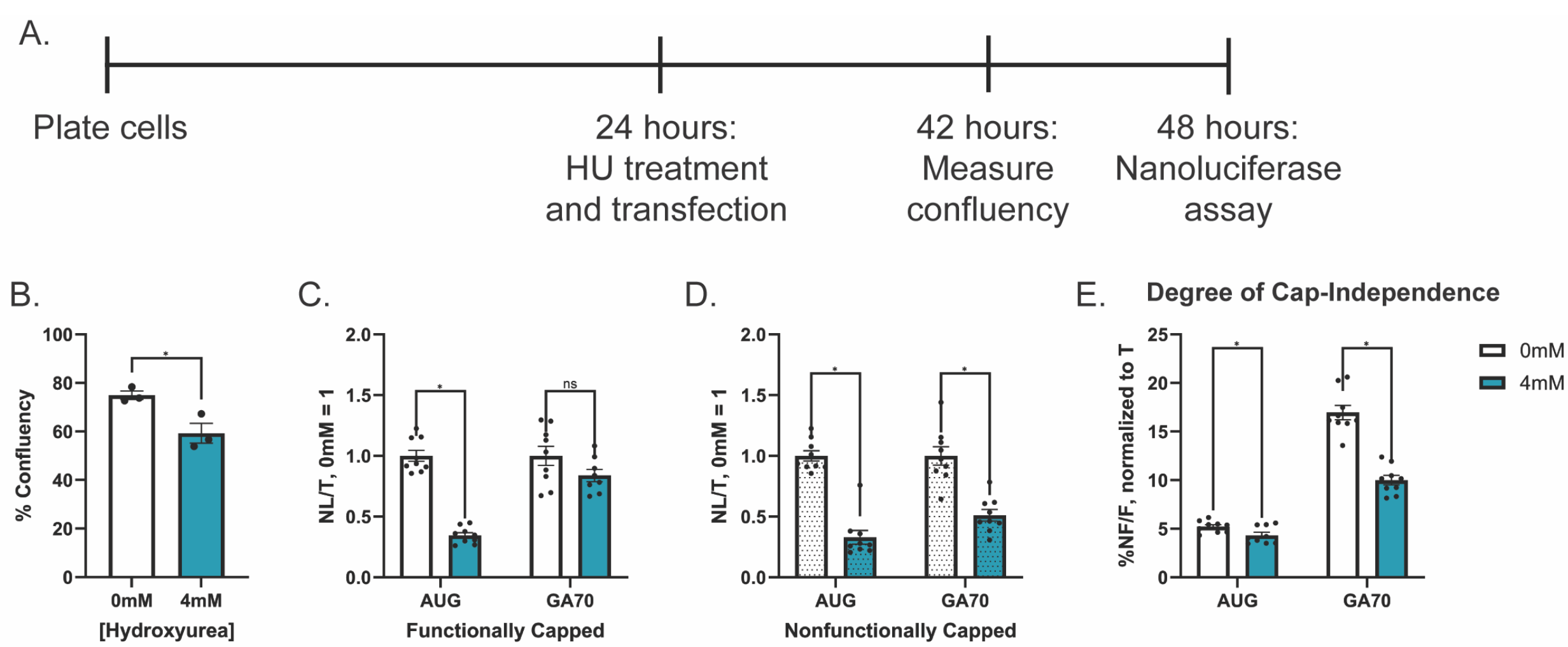

Figure S2: Cell division does not explain high cap-dependence of RAN translation. (A) Timeline of experiment. (B) Percent confluency of cells after treatment with hydroxyurea (HU) for 18 hours, n = 3 plates. (C-D) Relative expression of RAN reporters in HU or vehicle treated HEK293 cells after transfection with functionally capped (C) or nonfunctionally capped (D) mRNA for 24 hours. Data is expressed as NL signal normalized to titer, n = 9. (E) Degree of cap-independence of reporters after transfection into HU or vehicle treated HEK293 cells after transfection for 24 hours. Data is expressed as percent signal from nonfunctionally capped mRNA (NF) compared to the functionally capped mRNA (F), normalized to titer, n = 9. Graphs

represent mean ± SEM; (B) Two-tailed Student's t test with Welch's correction. (C-E) Multiple Holm–Šídák's two-tailed Student's t test with Welch's correction.*P < 0.05, **P<0.01, ***P < 0.001, ****P < 0.0001.

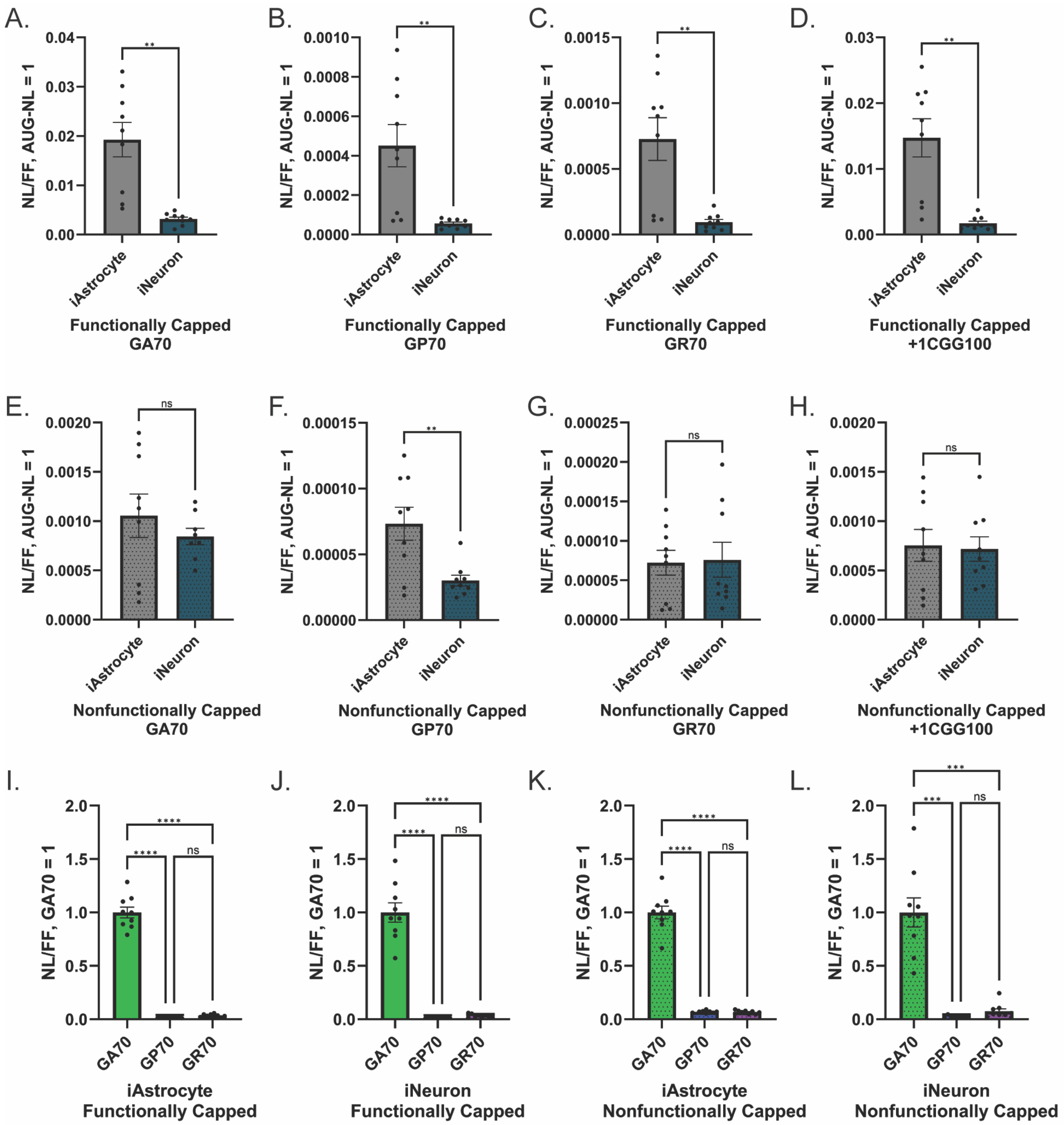

Figure S3: Expression of RAN reporters in human-derived cells. Relative expression of RAN reporters in iAstrocytes and iNeurons after transfection with functionally capped (A–D) or nonfunctionally capped (E–H) mRNA for 24 hours. Data is expressed as NL signal normalized to

FF and relative to functionally capped AUG-NL reporter expression, n = 12. (I–L) Relative expression of C9 RAN reporters in iAstrocytes and iNeurons after transfection with functionally capped (I–J) or nonfunctionally capped (K–L) mRNA for 24 hours. Data is expressed as NL signal normalized to FF and relative to GA70 reporter expression, n = 12. Graphs represent mean ± SEM; (A–H) Two-tailed Student's t test with Welch's correction. (I–L) Brown-Forsythe one-way ANOVA with Dunnet's multiple comparison correction. *P < 0.05, **P<0.01, ***P < 0.001, ****P < 0.0001.

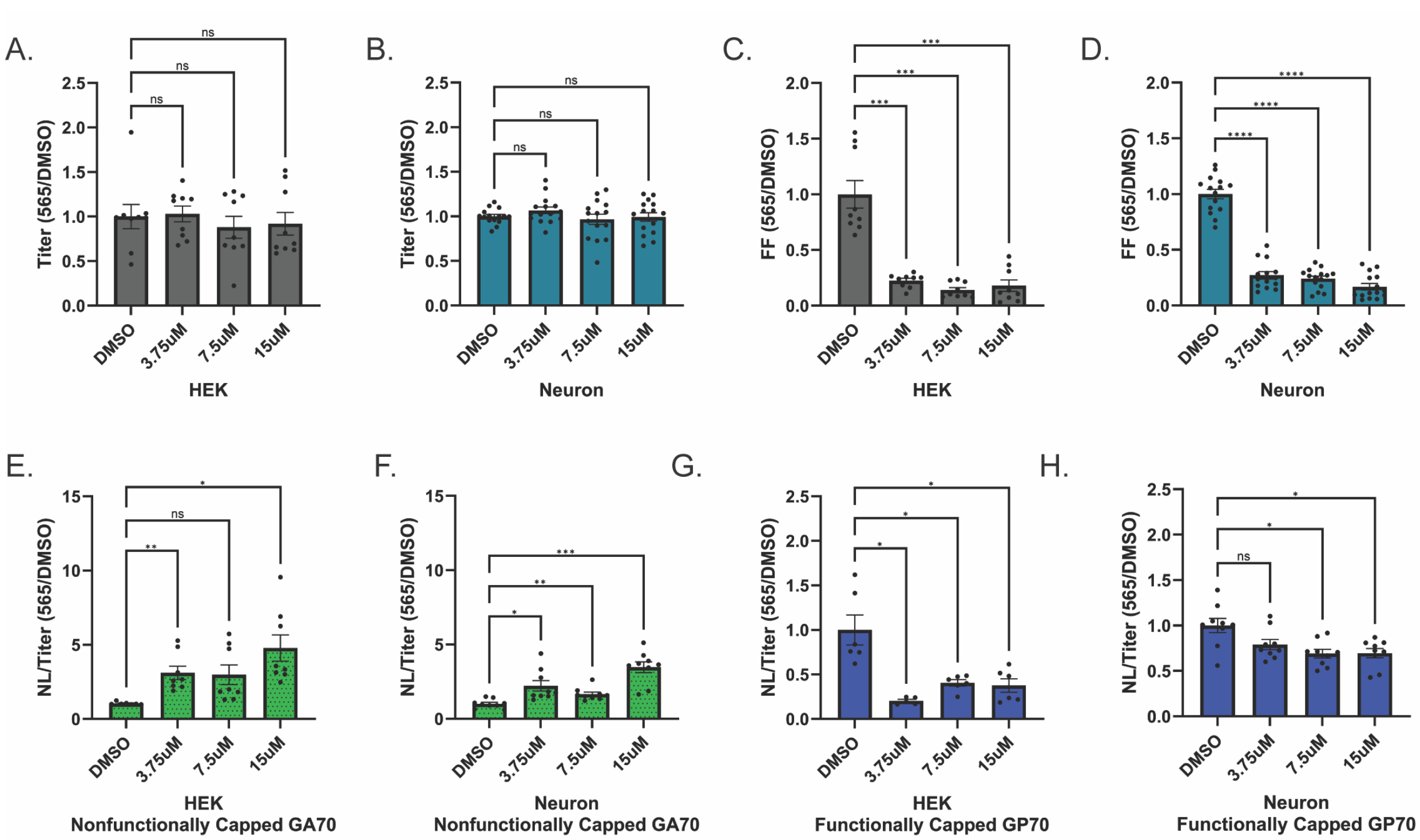

Figure S4: Effect of cap-binding factor eIF4E inhibition on non-canonical translation. (A–B) Cell viability measured by cell titer after 24 hours of treatment with 0 – 15μM eIF4E competitive inhibitor eFT-565 (565) in HEK293 cells (A) or primary rat hippocampal neurons (B) transfected with AUG-NL reporter 4.5 hours after treatment. Data expressed as titer signal relative to DMSO condition, n = 9 (A), n = 15 (B). (C–D) Effect on cotransfected firefly luciferase reporter signal after 24 hours of treatment with 0 – 15μM eFT-565 in HEK293 cells (C) or primary rat hippocampal neurons (D) transfected with AUG-NL reporter 4.5 hours after treatment. Data expressed as titer signal relative to DMSO condition, n = 9 (C), n = 15 (D). (E–F) Relative response of nonfunctionally capped GA70 to treatment with eFT-565. HEK293 cells (E) and rat

primary hippocampal neurons (F) were treated with 0 – 15µM eFT-565, transfected with nonfunctionally capped GA70 reporter 4.5 hours after treatment, and assayed for translation 19.5 hours later. Data is expressed as NL signal normalized to titer and relative to DMSO treated condition, n = 9. (G–H) Relative response of GP70 to treatment with eFT-565. HEK293 cells (G) and rat primary hippocampal neurons (H) were treated with 0 – 15µM eFT-565, transfected with functionally capped GP70 reporter 4.5 hours after treatment, and assayed for translation 19.5 hours later. Data is expressed as NL signal normalized to titer and relative to DMSO treated condition, n = 6. Graphs represent mean ± SEM; Brown-Forsythe one-way ANOVA with Dunnet's multiple comparison correction. *P < 0.05, **P<0.01, ***P < 0.001, ****P < 0.0001.

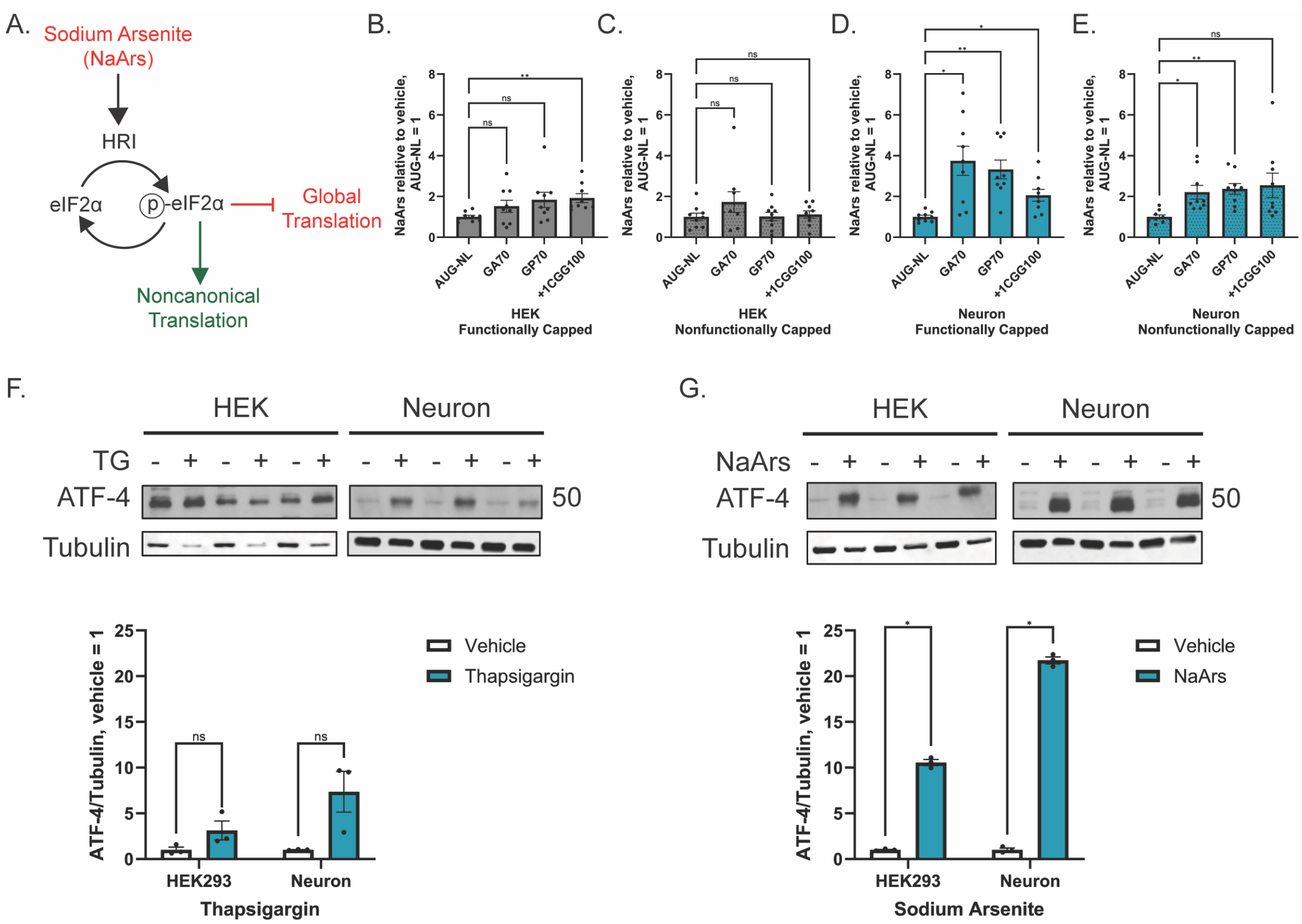

Figure S5: Cap-independent RAN translation is resistant to inhibition by sodium arsenite activation of the integrated stress response. (A) Schematic of the oxidative stress branch of the integrated stress response. (B–E) Relative expression of reporters in HEK293 cells (B-C) and

rat primary hippocampal neurons (D–E) treated with 20μM sodium arsenite (NaArs), transfected with functionally capped (B/D) or nonfunctionally capped (C/E) reporters, and assayed for translation 5 hours later. Data is expressed as ratio of NL signal normalized to titer and relative to AUG response, n = 9. (F-G) Western blot and quantification of HEK293 cells and rat primary hippocampal neurons treated with 2μM thapsigargin (F) or 20μM NaArs (G) for 5 hours. Blots were probed for ATF-4 as a marker of the integrated stress response. Graphs represent mean ± SEM; (B-E) Brown-Forsythe one-way ANOVA with Dunnet's multiple comparison correction. (F-G) Multiple Holm–Šídák's two-tailed Student's t test with Welch's correction. *P < 0.05, **P<0.01, ***P < 0.001, ****P < 0.0001.

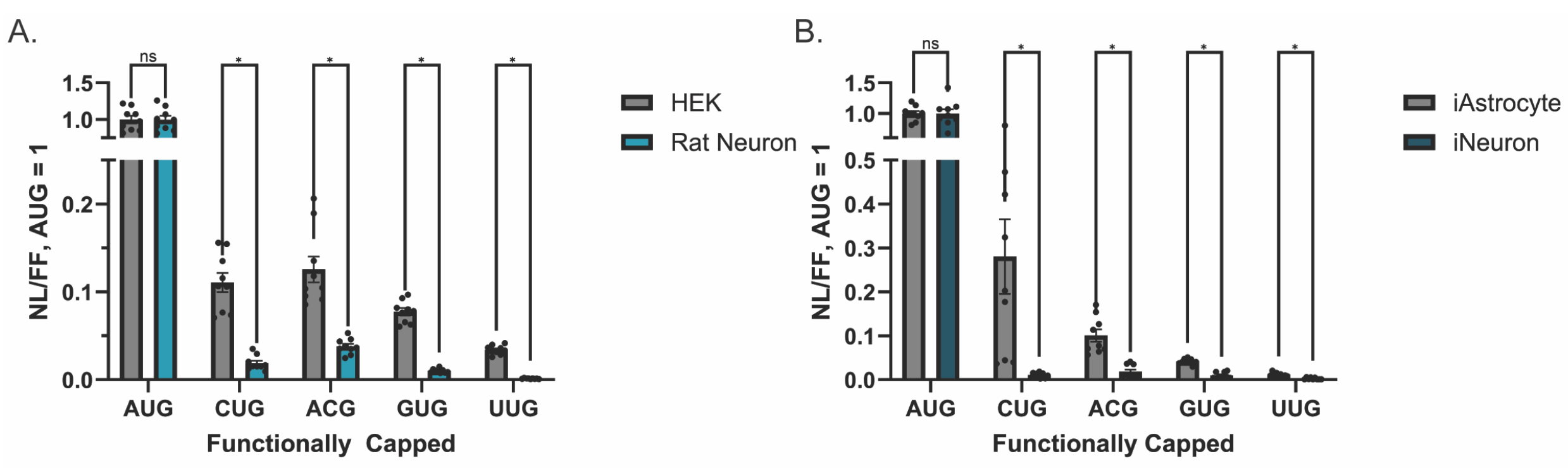

Figure S6: Start codon stringency across near cognate start codons is higher in neurons. Relative expression of near cognate codon reporters in HEK293 cells and rat primary hippocampal neurons (A) or iAstrocytes and iNeurons (B) after transfection with functionally capped mRNA for 24 hours. Data is expressed as NL signal normalized to FF with AUG-NL set to 1. Graphs represent mean ± SEM; Multiple Holm–Šídák's two-tailed Student's t test with Welch's correction. *P < 0.05, **P<0.01, ***P < 0.001, ****P < 0.0001.

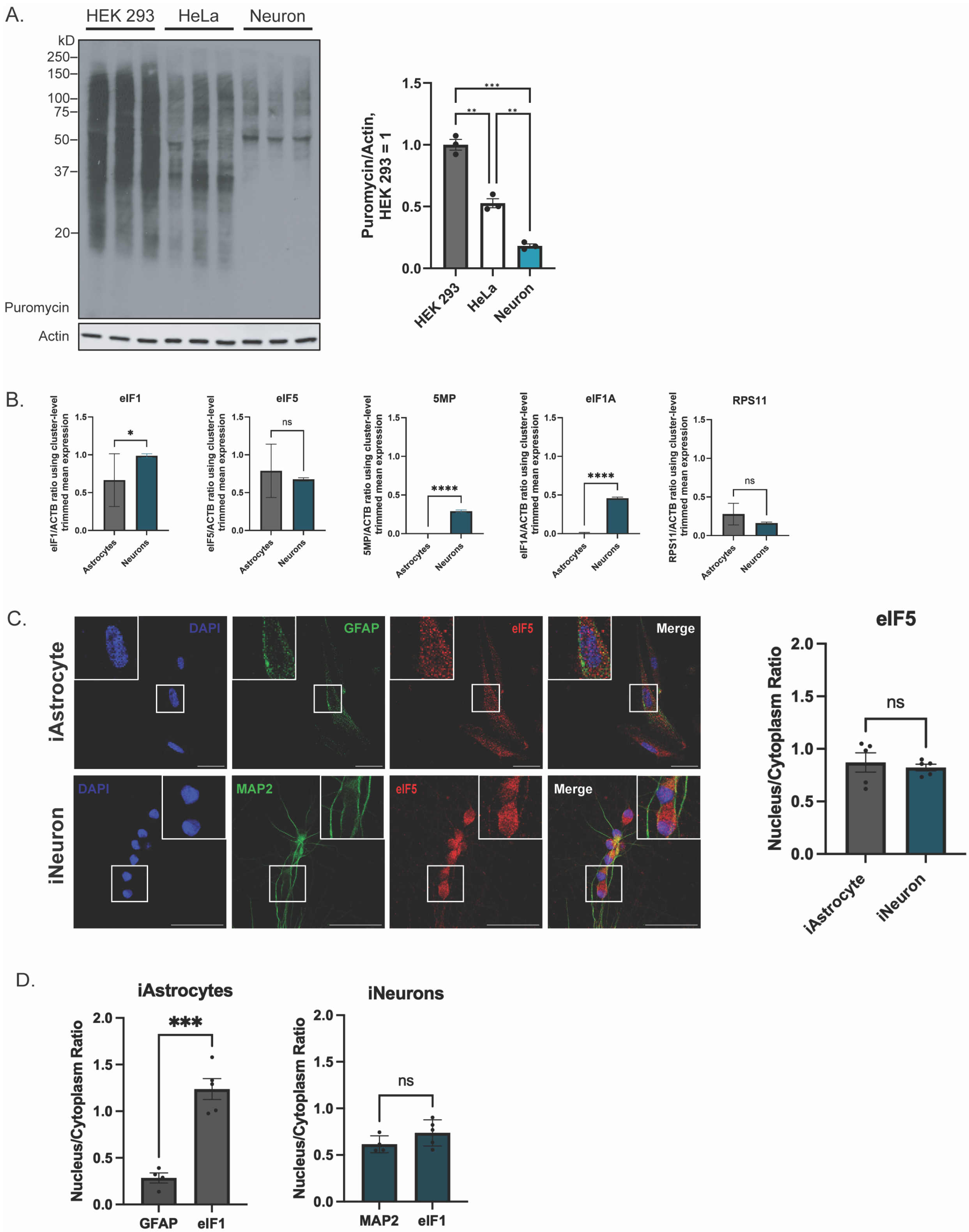
A.
HEK 293
HeLa
Neuron
kD
250
150
100
75
50
37
20
Puromycin
Actin
Puromycin/Actin, HEK 293 = 1
1.5
1.0
0.5
0.0
***
**
**
HEK 293
HeLa
Neuron
B.
eIF1
eIF5
5MP
eIF1A
RPS11
eIF1/ACTB ratio using cluster-level trimmed mean expression
eIF5/ACTB ratio using cluster-level trimmed mean expression
5MP/ACTB ratio using cluster-level trimmed mean expression
eIF1A/ACTB ratio using cluster-level trimmed mean expression
RPS11/ACTB ratio using cluster-level trimmed mean expression
*
ns
****
****
ns
Astrocytes
Neurons
C.
iAstrocyte
iNeuron
DAPI
GFAP
eIF5
Merge
MAP2
eIF5
Nucleus/Cytoplasm Ratio
2.0
1.5
1.0
0.5
0.0
ns
iAstrocyte
iNeuron
D.
iAstrocytes
iNeurons
Nucleus/Cytoplasm Ratio
***
ns
GFAP
eIF1
MAP2
eIF1

Figure S7: Initiation factor variation by cell type. (A) SUnSET assay and quantification of puromycin incorporation in HEK293 cells, HeLa cells and rat primary hippocampal neurons. Data is expressed as intensity in each condition normalized to actin (loading control), n = 3. (B) *In silico* quantification of initiation factor expression across cell types. The data utilized cluster-level trimmed means to compute ratios relative to ACTB. Transcriptomic clusters were grouped into Neurons, n = 226, and Astrocytes n = 6. (C) Representative ICC images and quantification of the nucleus to cytoplasm ratio for eIF5 in iAstrocytes and iNeurons. Scale bar = 50μm. Data is expressed as ratio of nuclear and cytoplasmic brightness of all cells in a single image with each n representing the mean of separate images from a given well, n = 5. (D) Quantification of nucleus to cytoplasm ratio of eIF1 compared to GFAP and MAP2 in representative ICC images of iAstrocytes and iNeurons, respectively, n = 5. Data is expressed as average trimmed-mean per group normalized to ACTB. Graphs represent mean ± SEM; (A) Brown-Forsythe one-way ANOVA with Dunnet's multiple comparison correction; (B) Kolmogorov-Smirnov test; (C, D) two-tailed Student's t test with Welch's correction. *P < 0.05, **P<0.01, ***P < 0.001, ****P < 0.0001.

Table S1: Reporter Sequences

**Name**	**Sequence from T7 to PspOMI cut site**
**GA70**	GGTGTGTGTTTTTGTTTTTCCCACCCTCTCTCCCCACTACTTGCTCTCACAGTACTC<br>GCTGAGGGTGAACAAGAAAAGACCTGATAAAGATTAACCAGAAGAAAACAAGG<br>AGGGAAACAACCGCAGCCTGTAGCAAGCTCTGGAACTCAGGAGTCGCGCGCTA<br>GCGGCCGGGGCCGGGGCCGGGGCCGGGGCCGGGGCCGGGGCCGGGGCCGGG<br>GCCGGGGCCGGGGCCGGGGCCGGGGCCGGGGCAGGGGCCGGGGCCGGGGCC<br>GGGGCCGGGGCCGGGGCCGGGGCCGGGGCCGGGGCCGGGGCCGGGGCCGGG<br>GCCGGGGCCGGGGCCGGGGCCGGGGCCGGGGCCGGGGCCGGGGCCGGGGCC<br>GGGGCCGGGGCCGGGGCCGGGGCCGGGGCCGGGGCCGGGGCCGGGGCCGGG<br>GCCGGGGCCGGGGCCGGGGCCGGGGCCGGGGCCGGGGCCGGGGCCGGGGCC<br>GGGGCCGGGGCCGGGGCCGGGGCCGGGGCCGGGGCCGGGGCCGGGGCCGGG<br>GCCGGGGCCGGGGCCGGGGCCGGGGCCGGGGCCGGGGCCGGGGCCGGGGCC<br>GGGGCCGGGGCCGGGGCCGGTGGGGTCTTCACACTCGAAGATTTCGTTGGGGA<br>CTGGCGACAGACAGCCGGCTACAACCTGGACCAAGTCCTTGAACAGGGAGGTGT<br>GTCCAGTTTGTTTCAGAATCTCGGGGTGTCCGTAACTCCGATCCAAAGGATTGTC<br>CTGAGCGGTGAAAATGGGCTGAAGATCGACATCCATGTCATCATCCCGTATGAA<br>GGTCTGAGCGGCGACCAAATGGGCCAGATCGAAAAAATTTTTAAGGTGGTGTAC<br>CCTGTGGATGATCATCACTTTAAGGTGATCCTGCACTATGGCACACTGGTAATCG<br>ACGGGGTTACGCCGAACATGATCGACTATTTCGGACGGCCCTATGAAGGCATCG<br>CCGTGTTCGACGGCAAAAAGATCACTGTAACAGGGACCCTGTGGAACGGCAACA

	AAATTATCGACGAGCGCCTGATCAACCCCGACGGCTCCCTGCTGTTCCGAGTAAC<br>CATCAACGGAGTGACCGGCTGGCGGCTGTGCGAACGCATTCTGGCGGACTACAA<br>AGACCATGACGGTGATTATAAAGATCATGACATCGATTACAAGGATGACGATGA<br>CAAGTAAGGCCGCGACTCGAGAG
**GP70**	GGTGTGTGTTTTTGTTTTTCCCACCCTCTCTCCCCACTACTTGCTCTCACAGTACTC<br>GCTGAGGGTGAACAAGAAAAGACCTGATAAAGATTAACCAGAAGAAAACAAGG<br>AGGGAAACAACCGCAGCCTGTAGCAAGCTCTGGAACTCAGGAGTCGCGCGCTA<br>GCGGCCGGGGCCGGGGCCGGGGCCGGGGCCGGGGCCGGGGCCGGGGCCGGG<br>GCCGGGGCCGGGGCCGGGGCCGGGGCCGGGGCAGGGGCCGGGGCCGGGGCC<br>GGGGCCGGGGCCGGGGCCGGGGCCGGGGCCGGGGCCGGGGCCGGGGCCGGG<br>GCCGGGGCCGGGGCCGGGGCCGGGGCCGGGGCCGGGGCCGGGGCCGGGGCC<br>GGGGCCGGGGCCGGGGCCGGGGCCGGGGCCGGGGCCGGGGCCGGGGCCGGG<br>GCCGGGGCCGGGGCCGGGGCCGGGGCCGGGGCCGGGGCCGGGGCCGGGGCC<br>GGGGCCGGGGCCGGGGCCGGGGCCGGGGCCGGGGCCGGGGCCGGGGCCGGG<br>GCCGGGGCCGGGGCCGGGGCCGGGGCCGGGGCCGGGGCCGGGGCCGGGGCC<br>GGGGCCGGGGCCGGGGCCGGTTGGGGTCTTCACACTCGAAGATTTCGTTGGGG<br>ACTGGCGACAGACAGCCGGCTACAACCTGGACCAAGTCCTTGAACAGGGAGGT<br>GTGTCCAGTTTGTTTCAGAATCTCGGGGTGTCCGTAACTCCGATCCAAAGGATTG<br>TCCTGAGCGGTGAAAATGGGCTGAAGATCGACATCCATGTCATCATCCCGTATGA<br>AGGTCTGAGCGGCGACCAAATGGGCCAGATCGAAAAAATTTTTAAGGTGGTGTA<br>CCCTGTGGATGATCATCACTTTAAGGTGATCCTGCACTATGGCACACTGGTAATC<br>GACGGGGTTACGCCGAACATGATCGACTATTTCGGACGGCCCTATGAAGGCATC<br>GCCGTGTTCGACGGCAAAAAGATCACTGTAACAGGGACCCTGTGGAACGGCAAC<br>AAAATTATCGACGAGCGCCTGATCAACCCCGACGGCTCCCTGCTGTTCCGAGTAA<br>CCATCAACGGAGTGACCGGCTGGCGGCTGTGCGAACGCATTCTGGCGGACTACA<br>AAGACCATGACGGTGATTATAAAGATCATGACATCGATTACAAGGATGACGATG<br>ACAAGTAAGGCCGCGACTCGAGAG
**GR70**	GGTGTGTGTTTTTGTTTTTCCCACCCTCTCTCCCCACTACTTGCTCTCACAGTACTC<br>GCTGAGGGTGAACAAGAAAAGACCTGATAAAGATTAACCAGAAGAAAACAAGG<br>AGGGAAACAACCGCAGCCTGTAGCAAGCTCTGGAACTCAGGAGTCGCGCGCTA<br>GCGGCCGGGGCCGGGGCCGGGGCCGGGGCCGGGGCCGGGGCCGGGGCCGGG<br>GCCGGGGCCGGGGCCGGGGCCGGGGCCGGGGCAGGGGCCGGGGCCGGGGCC<br>GGGGCCGGGGCCGGGGCCGGGGCCGGGGCCGGGGCCGGGGCCGGGGCCGGG<br>GCCGGGGCCGGGGCCGGGGCCGGGGCCGGGGCCGGGGCCGGGGCCGGGGCC<br>GGGGCCGGGGCCGGGGCCGGGGCCGGGGCCGGGGCCGGGGCCGGGGCCGGG<br>GCCGGGGCCGGGGCCGGGGCCGGGGCCGGGGCCGGGGCCGGGGCCGGGGCC<br>GGGGCCGGGGCCGGGGCCGGGGCCGGGGCCGGGGCCGGGGCCGGGGCCGGG<br>GCCGGGGCCGGGGCCGGGGCCGGGGCCGGGGCCGGGGCCGGGGCCGGGGCC<br>GGGGCCGGGGCCGGGGCCGGTTTGGGGTCTTCACACTCGAAGATTTCGTTGGGG<br>ACTGGCGACAGACAGCCGGCTACAACCTGGACCAAGTCCTTGAACAGGGAGGT<br>GTGTCCAGTTTGTTTCAGAATCTCGGGGTGTCCGTAACTCCGATCCAAAGGATTG<br>TCCTGAGCGGTGAAAATGGGCTGAAGATCGACATCCATGTCATCATCCCGTATGA<br>AGGTCTGAGCGGCGACCAAATGGGCCAGATCGAAAAAATTTTTAAGGTGGTGTA<br>CCCTGTGGATGATCATCACTTTAAGGTGATCCTGCACTATGGCACACTGGTAATC

	GACGGGGTTACGCCGAACATGATCGACTATTTCGGACGGCCCTATGAAGGCATC<br>GCCGTGTTCGACGGCAAAAAGATCACTGTAACAGGGACCCTGTGGAACGGCAAC<br>AAAATTATCGACGAGCGCCTGATCAACCCCGACGGCTCCCTGCTGTTCCGAGTAA<br>CCATCAACGGAGTGACCGGCTGGCGGCTGTGCGAACGCATTCTGGCGGACTACA<br>AAGACCATGACGGTGATTATAAAGATCATGACATCGATTACAAGGATGACGATG<br>ACAAGTAAGGCCGCGACTCGAGAG
**CCC-GA70**	GGTGTGTGTTTTTGTTTTTCCCACCCTCTCTCCCCACTACTTGCTCTCACAGTACTC<br>GCTGAGGGTGAACAAGAAAAGACCTGATAAAGATTAACCAGAAGAAAACAAGG<br>AGGGAAACAACCGCAGCCTGTAGCAAGCTCCCGAACTCAGGAGTCGCGCGCTA<br>GCGGCCGGGGCCGGGGCCGGGGCCGGGGCCGGGGCCGGGGCCGGGGCCGGG<br>GCCGGGGCCGGGGCCGGGGCCGGGGCCGGGGCAGGGGCCGGGGCCGGGGCC<br>GGGGCCGGGGCCGGGGCCGGGGCCGGGGCCGGGGCCGGGGCCGGGGCCGGG<br>GCCGGGGCCGGGGCCGGGGCCGGGGCCGGGGCCGGGGCCGGGGCCGGGGCC<br>GGGGCCGGGGCCGGGGCCGGGGCCGGGGCCGGGGCCGGGGCCGGGGCCGGG<br>GCCGGGGCCGGGGCCGGGGCCGGGGCCGGGGCCGGGGCCGGGGCCGGGGCC<br>GGGGCCGGGGCCGGGGCCGGGGCCGGGGCCGGGGCCGGGGCCGGGGCCGGG<br>GCCGGGGCCGGGGCCGGGGCCGGGGCCGGGGCCGGGGCCGGGGCCGGGGCC<br>GGGGCCGGGGCCGGGGCCGGTGGGGTCTTCACACTCGAAGATTTCGTTGGGGA<br>CTGGCGACAGACAGCCGGCTACAACCTGGACCAAGTCCTTGAACAGGGAGGTGT<br>GTCCAGTTTGTTTCAGAATCTCGGGGTGTCCGTAACTCCGATCCAAAGGATTGTC<br>CTGAGCGGTGAAAATGGGCTGAAGATCGACATCCATGTCATCATCCCGTATGAA<br>GGTCTGAGCGGCGACCAAATGGGCCAGATCGAAAAAATTTTTAAGGTGGTGTAC<br>CCTGTGGATGATCATCACTTTAAGGTGATCCTGCACTATGGCACACTGGTAATCG<br>ACGGGGTTACGCCGAACATGATCGACTATTTCGGACGGCCCTATGAAGGCATCG<br>CCGTGTTCGACGGCAAAAAGATCACTGTAACAGGGACCCTGTGGAACGGCAACA<br>AAATTATCGACGAGCGCCTGATCAACCCCGACGGCTCCCTGCTGTTCCGAGTAAC<br>CATCAACGGAGTGACCGGCTGGCGGCTGTGCGAACGCATTCTGGCGGACTACAA<br>AGACCATGACGGTGATTATAAAGATCATGACATCGATTACAAGGATGACGATGA<br>CAAGTAAGGCCGCGACTCGAGAG
**AUG-GA70**	GGTGTGTGTTTTTGTTTTTCCCACCCTCTCTCCCCACTACTTGCTCTCACAGTACTC<br>GCTGAGGGTGAACAAGAAAAGACCTGATAAAGATTAACCAGAAGAAAACAAGG<br>AGGGAAACAACCGCAGCCTGTAGCAAGCTATGGAACTCAGGAGTCGCGCGCTA<br>GCGGCCGGGGCCGGGGCCGGGGCCGGGGCCGGGGCCGGGGCCGGGGCCGGG<br>GCCGGGGCCGGGGCCGGGGCCGGGGCCGGGGCAGGGGCCGGGGCCGGGGCC<br>GGGGCCGGGGCCGGGGCCGGGGCCGGGGCCGGGGCCGGGGCCGGGGCCGGG<br>GCCGGGGCCGGGGCCGGGGCCGGGGCCGGGGCCGGGGCCGGGGCCGGGGCC<br>GGGGCCGGGGCCGGGGCCGGGGCCGGGGCCGGGGCCGGGGCCGGGGCCGGG<br>GCCGGGGCCGGGGCCGGGGCCGGGGCCGGGGCCGGGGCCGGGGCCGGGGCC<br>GGGGCCGGGGCCGGGGCCGGGGCCGGGGCCGGGGCCGGGGCCGGGGCCGGG<br>GCCGGGGCCGGGGCCGGGGCCGGGGCCGGGGCCGGGGCCGGGGCCGGGGCC<br>GGGGCCGGGGCCGGGGCCGGTGGGGTCTTCACACTCGAAGATTTCGTTGGGGA<br>CTGGCGACAGACAGCCGGCTACAACCTGGACCAAGTCCTTGAACAGGGAGGTGT<br>GTCCAGTTTGTTTCAGAATCTCGGGGTGTCCGTAACTCCGATCCAAAGGATTGTC<br>CTGAGCGGTGAAAATGGGCTGAAGATCGACATCCATGTCATCATCCCGTATGAA

	GGTCTGAGCGGCGACCAAATGGGCCAGATCGAAAAAATTTTTAAGGTGGTGTAC<br>CCTGTGGATGATCATCACTTTAAGGTGATCCTGCACTATGGCACACTGGTAATCG<br>ACGGGGTTACGCCGAACATGATCGACTATTTCGGACGGCCCTATGAAGGCATCG<br>CCGTGTTCGACGGCAAAAAGATCACTGTAACAGGGACCCTGTGGAACGGCAACA<br>AAATTATCGACGAGCGCCTGATCAACCCCGACGGCTCCCTGCTGTTCCGAGTAAC<br>CATCAACGGAGTGACCGGCTGGCGGCTGTGCGAACGCATTCTGGCGGACTACAA<br>AGACCATGACGGTGATTATAAAGATCATGACATCGATTACAAGGATGACGATGA<br>CAAGTAAGGCCGCGACTCGAGAG
**+1CGG100**	GGGAGACCCAAGCTGGCTAGCGTTTAAACTTAAGCTTGGTACCGAGCTCGGATCCA<br>CTAGTCCAGTGTGGTGGAATTCGTTAACAGATCTGCTCAGCTCCGTTTCGGTTTCACT<br>TCCGGTGGAGGGCCGCCTCTGAGCGGGCGGCGGGCCGACGGCGAGCGCGGGCG<br>GCGGCGGTGACGGAGGCGCCGCTGCCAGGGGGCGTGCGGCAGCGCGGCGGCGG<br>CGGCGGCGGCGGCGGAGGCGGCGGCGGCGGCGGCGGCGGCGGCGGCGGCGGC<br>GGCGGCGGCGGCGGCGGCGGCGGCGGCGGCGGCGGCGGCGGCGGCGGCGGCG<br>GCGGCGGCGGCGGCGGCGGCGGCGGCGGCGGCGGCGGCGGCGGCGGCGGCGG<br>CGGCGGCGGCGGCGGCGGCGGCGGCGGCGGCGGCGGCGGCGGCGGCGGCGGC<br>GGCGGCGGCGGCGGCGGCGGCGGCGGCGGCGGCGGCGGCGGCGGCGGCGGCG<br>GCGGCGGCGGCGGCGGCGGCGGCGGCGGCGGCGGCTGGGCCTCGAGGATATCA<br>AGATCTGGCCTCGGCGGCCAAGCTTGGCAATCCGGTACTGTTGGTAAAGCCACCGG<br>GGTCTTCACACTCGAAGATTTCGTTGGGGACTGGCGACAGACAGCCGGCTACAAC<br>CTGGACCAAGTCCTTGAACAGGGAGGTGTGTCCAGTTTGTTTCAGAATCTCGGGGT<br>GTCCGTAACTCCGATCCAAAGGATTGTCCTGAGCGGTGAAAATGGGCTGAAGATCG<br>ACATCCATGTCATCATCCCGTATGAAGGTCTGAGCGGCGACCAAATGGGCCAGATC<br>GAAAAAATTTTTAAGGTGGTGTACCCTGTGGATGATCATCACTTTAAGGTGATCCTG<br>CACTATGGCACACTGGTAATCGACGGGGTTACGCCGAACATGATCGACTATTTCGG<br>ACGGCCGTATGAAGGCATCGCCGTGTTCGACGGCAAAAAGATCACTGTAACAGGG<br>ACCCTGTGGAACGGCAACAAAATTATCGACGAGCGCCTGATCAACCCCGACGGCTC<br>CCTGCTGTTCCGAGTAACCATCAACGGAGTGACCGGCTGGCGGCTGTGCGAACGC<br>ATTCTGGCGGACTACAAAGACCATGACGGTGATTATAAAGATCATGACATCGATTAC<br>AAGGATGACGATGACAAGTAAGGCCGCGACTCTAGAG
**AUG-NL**	GGGAGACCCAAGCTGGCTAGCGTTTAAACTTAAGCTTGGCAATCCGGTACTGTTGG<br>TAAATAAGCCACCATGGTCTTCACACTCGAAGATTTCGTTGGGGACTGGCGACAGA<br>CAGCCGGCTACAACCTGGACCAAGTCCTTGAACAGGGAGGTGTGTCCAGTTTGTTT<br>CAGAATCTCGGGGTGTCCGTAACTCCGATCCAAAGGATTGTCCTGAGCGGTGAAAA<br>TGGGCTGAAGATCGACATCCATGTCATCATCCCGTATGAAGGTCTGAGCGGCGACC<br>AAATGGGCCAGATCGAAAAAATTTTTAAGGTGGTGTACCCTGTGGATGATCATCACT<br>TTAAGGTGATCCTGCACTATGGCACACTGGTAATCGACGGGGTTACGCCGAACATG<br>ATCGACTATTTCGGACGGCCGTATGAAGGCATCGCCGTGTTCGACGGCAAAAAGAT<br>CACTGTAACAGGGACCCTGTGGAACGGCAACAAAATTATCGACGAGCGCCTGATC<br>AACCCCGACGGCTCCCTGCTGTTCCGAGTAACCATCAACGGAGTGACCGGCTGGCG<br>GCTGTGCGAACGCATTCTGGCGGACTACAAAGACCATGACGGTGATTATAAAGATC<br>ATGACATCGATTACAAGGATGACGATGACAAGTAAGGCCGCGACTCTAGAG
**CUG-NL**	GGGAGACCCAAGCTGGCTAGCGTTTAAACTTAAGCTTGGCAATCCGGTACTGTTGG<br>TAAATAAGCCACCCTGGTCTTCACACTCGAAGATTTCGTTGGGGACTGGCGACAGA

	CAGCCGGCTACAACCTGGACCAAGTCCTTGAACAGGGAGGTGTGTCCAGTTTGTTT<br>CAGAATCTCGGGGTGTCCGTAACTCCGATCCAAAGGATTGTCCTGAGCGGTGAAAA<br>TGGGCTGAAGATCGACATCCATGTCATCATCCCGTATGAAGGTCTGAGCGGCGACC<br>AAATGGGCCAGATCGAAAAAATTTTTAAGGTGGTGTACCCTGTGGATGATCATCACT<br>TTAAGGTGATCCTGCACTATGGCACACTGGTAATCGACGGGGTTACGCCGAACATG<br>ATCGACTATTTCGGACGGCCGTATGAAGGCATCGCCGTGTTCGACGGCAAAAAGAT<br>CACTGTAACAGGGACCCTGTGGAACGGCAACAAAATTATCGACGAGCGCCTGATC<br>AACCCCGACGGCTCCCTGCTGTTCCGAGTAACCATCAACGGAGTGACCGGCTGGCG<br>GCTGTGCGAACGCATTCTGGCGGACTACAAAGACCATGACGGTGATTATAAAGATC<br>ATGACATCGATTACAAGGATGACGATGACAAGTAAGGCCGCGACTCTAGAG
**eIF5**	AGGGAGACCCAAGCTGGCTAGTTAAGCTTGGTACCGAGCTCGGATCCAGTACCCTT<br>CACCATGTCTGTCAATGTCAACCGCAGCGTGTCAGACCAGTTCTATCGCTACAAGAT<br>GCCCCGTCTGATTGCCAAGGTTGAGGGCAAAGGCAATGGAATCAAGACAGTTATA<br>GTCAACATGGTTGACGTTGCAAAGGCGCTTAATCGGCCTCCAACGTATCCCACCAA<br>ATATTTTGGTTGTGAGCTGGGAGCACAGACCCAGTTTGATGTTAAGAATGACCGTTA<br>CATTGTCAATGGATCTCATGAGGCGAATAAGCTGCAAGACATGTTGGATGGATTCAT<br>TAAAAAATTTGTTCTCTGTCCTGAATGTGAGAATCCTGAAACAGATTTGCATGTCAAT<br>CCAAAGAAGCAAACAATAGGTAATTCTTGTAAAGCCTGTGGCTATCGAGGCATGCT<br>TGACACACATCATAAACTCTGCACATTCATTCTCAAAAACCCACCTGAGAATAGTGA<br>CAGTGGTACAGGAAAGAAAGAAAAAGAAAAGAAAAACAGAAAGGGCAAAGACA<br>AGGAAAATGGCTCCGTATCCAGCAGTGAGACACCACCACCACCACCACCACCAAA<br>TGAAATTAATCCTCCTCCACATACAATGGAAGAAGAGGAGGATGATGACTGGGGAG<br>AAGATACAACTGAGGAAGCTCAAAGGCGTCGAATGGATGAAATCAGTGACCATGC<br>AAAAGTTCTGACACTCAGTGATGATTTGGAAAGAACAATTGAGGAGAGGGTCAATA<br>TCCTCTTTGATTTTGTTAAGAAAAAGAAAGAAGAGGGTGTTATTGATTCATCTGACA<br>AAGAAATCGTTGCTGAAGCAGAAAGACTGGATGTAAAAGCCATGGGCCCTCTTGTT<br>CTAACTGAAGTTCTTTTTAATGAGAAGATTAGAGAACAGATTAAGAAATACAGGCG<br>CCATTTCCTACGATTTTGTCACAACAACAAAAAAGCCCAACGGTACCTTCTTCATGG<br>TTTGGAGTGTGTGGTAGCAATGCATCAAGCTCAGCTTATCTCCAAGATTCCACATATC<br>TTGAAGGAGATGTACGATGCAGACCTTTTAGAAGAAGAGGTCATCATCAGCTGGTC<br>GGAAAAGGCCTCTAAGAAATATGTCTCCAAAGAACTTGCCAAAGAGATTCGTGTCA<br>AAGCAGAACCATTTATAAAATGGTTGAAGGAGGCAGAGGAAGAATCTTCTGGTGG<br>CGAAGAAGAAGATGAAGATGAGAACATTGAGGTGGTGTATTCGAAGGCTGCCAGT<br>GTACCGAAAGTTGAGACTGTAAAGTCAGACAACAAGGATGACGACATCGATATTGA<br>TGCCATAAGGGTCAAGACAATTCTGCAGATATCCAGCACAGTGGCGGCCGCTCGAG<br>TCTAGAGGGCCCGCGGTTCGAAGGTAAGCCTATCCCTAACCCTCTCCTCGGTCTCGA<br>TTCTACGCGTACCGGTCATCATCACCATCACCATTGAGTTT

Name	Sequence from T7 to PmeI cut site
**FF**	GGGAGACCCAAGCTGGCTAGCTGAATTCCTTAAGCTTGGCAATCCGGTACTGTTGG<br>TAAAGCCACCATGGAAGATGCCAAAAACATTAAGAAGGGCCCAGCGCCATTCTAC<br>CCACTCGAAGACGGGACCGCCGGCGAGCAGCTGCACAAAGCCATGAAGCGCTAC<br>GCCCTGGTGCCCGGCACCATCGCCTTTACCGACGCACATATCGAGGTGGACATTAC

	CTACGCCGAGTACTTCGAGATGAGCGTTCGGCTGGCAGAAGCTATGAAGCGCTATG<br>GGCTGAATACAAACCATCGGATCGTGGTGTGCAGCGAGAATAGCTTGCAGTTCTTC<br>ATGCCCGTGTTGGGTGCCCTGTTCATCGGTGTGGCTGTGGCCCCAGCTAACGACATC<br>TACAACGAGCGCGAGCTGCTGAACAGCATGGGCATCAGCCAGCCCACCGTCGTATT<br>CGTGAGCAAGAAAGGGCTGCAAAAGATCCTCAACGTGCAAAAGAAGCTACCGATC<br>ATACAAAAGATCATCATCATGGATAGCAAGACCGACTACCAGGGCTTCCAAAGCAT<br>GTACACCTTCGTGACTTCCCATTTGCCACCCGGCTTCAACGAGTACGACTTCGTGCC<br>CGAGAGCTTCGACCGGGACAAAACCATCGCCCTGATCATGAACAGTAGTGGCAGT<br>ACCGGATTGCCCAAGGGCGTAGCCCTACCGCACCGCACCGCTTGTGTCCGATTCAG<br>TCATGCCCGCGACCCCATCTTCGGCAACCAGATCATCCCCGACACCGCTATCCTCAG<br>CGTGGTGCCATTTCACCACGGCTTCGGCATGTTCACCACGCTGGGCTACTTGATCTG<br>CGGCTTTCGGGTCGTGCTCATGTACCGCTTCGAGGAGGAGCTATTCTTGCGCAGCTT<br>GCAAGACTATAAGATTCAATCTGCCCTGCTGGTGCCCACACTATTTAGCTTCTTCGCT<br>AAGAGCACTCTCATCGACAAGTACGACCTAAGCAACTTGCACGAGATCGCCAGCG<br>GCGGGGCGCCGCTCAGCAAGGAGGTAGGTGAGGCCGTGGCCAAACGCTTCCACC<br>TACCAGGCATCCGCCAGGGCTACGGCCTGACAGAAACAACCAGCGCCATTCTGAT<br>CACCCCCGAAGGGGACGACAAGCCTGGCGCAGTAGGCAAGGTGGTGCCCTTCTTC<br>GAGGCTAAGGTGGTGGACTTGGACACCGGTAAGACACTGGGTGTGAACCAGCGCG<br>GCGAGCTGTGCGTCCGTGGCCCCATGATCATGAGCGGCTACGTTAACAACCCCGAG<br>GCTACAAACGCTCTCATCGACAAGGACGGCTGGCTGCACAGCGGCGACATCGCCT<br>ACTGGGACGAGGACGAGCACTTCTTCATCGTGGACCGGCTGAAGAGCCTGATCAA<br>ATACAAGGGCTACCAGGTAGCCCCAGCCGAACTGGAGAGCATCCTGCTGCAACAC<br>CCCAACATCTTCGACGCCGGGGTCGCCGGCCTGCCCGACGACGATGCCGGCGAGC<br>TGCCCGCCGCAGTCGTCGTGCTGGAACACGGTAAAACCATGACCGAGAAGGAGAT<br>CGTGGACTATGTGGCCAGCCAGGTTACAACCGCCAAGAAGCTGCGCGGTGGTGTT<br>GTGTTCGTGGACGAGGTGCCTAAAGGACTGACCGGCAAGTTGGACGCCCGCAAGA<br>TCCGCGAGATTCTCATTAAGGCCAAGAAGGGCGGCAAGATCGCCGTGTAATAATTC<br>TAGAGGGCCCGTTTA
